# Neurophysiologic Characterization of Resting State Connectivity Abnormalities in Schizophrenia Patients

**DOI:** 10.3389/fpsyt.2020.608154

**Published:** 2020-11-27

**Authors:** Daisuke Koshiyama, Makoto Miyakoshi, Kumiko Tanaka-Koshiyama, Yash B. Joshi, Juan L. Molina, Joyce Sprock, David L. Braff, Gregory A. Light

**Affiliations:** ^1^Department of Psychiatry, University of California, San Diego, La Jolla, CA, United States; ^2^Swartz Center for Neural Computation, University of California, San Diego, La Jolla, CA, United States; ^3^VISN-22 Mental Illness, Research, Education and Clinical Center, VA San Diego Healthcare System, San Diego, CA, United States

**Keywords:** resting-state electroencephalography (EEG), effective connectivity, schizophrenia, source level analysis, biomarker, temporal cortex, frontal cortex

## Abstract

**Background:** Patients with schizophrenia show abnormal spontaneous oscillatory activity in scalp-level electroencephalographic (EEG) responses across multiple frequency bands. While oscillations play an essential role in the transmission of information across neural networks, few studies have assessed the frequency-specific dynamics across cortical source networks at rest. Identification of the neural sources and their dynamic interactions may improve our understanding of core pathophysiologic abnormalities associated with the neuropsychiatric disorders.

**Methods:** A novel multivector autoregressive modeling approach for assessing effective connectivity among cortical sources was developed and applied to resting-state EEG recordings obtained from *n* = 139 schizophrenia patients and *n* = 126 healthy comparison subjects.

**Results:** Two primary abnormalities in resting-state networks were detected in schizophrenia patients. The first network involved the middle frontal and fusiform gyri and a region near the calcarine sulcus. The second network involved the cingulate gyrus and the Rolandic operculum (a region that includes the auditory cortex).

**Conclusions:** Schizophrenia patients show widespread patterns of hyper-connectivity across a distributed network of the frontal, temporal, and occipital brain regions. Results highlight a novel approach for characterizing alterations in connectivity in the neuropsychiatric patient populations. Further mechanistic characterization of network functioning is needed to clarify the pathophysiology of neuropsychiatric and neurological diseases.

## Introduction

Neurophysiologic abnormalities are commonly studied in patients with schizophrenia in response to experimental stimuli, cognitive, tasks, and even at rest. Neural oscillations play an essential role in cortico-cortical transmission and the integration of information across neural networks supporting critical brain functions, including perception, attention, and other higher-order cognitive functions (1–7).

Neural oscillations can be measured in the scalp electroencephalogram *via* a variety of analytic and experimental settings [e.g., spontaneous, evoked, induced, and emitted (8–10)], which have productively resulted in the identification of abnormalities across a broad range of conditions in schizophrenia patients. Task-related (i.e., evoked and induced) high frequency oscillatory abnormalities in schizophrenia patients, especially for gamma band oscillations (i.e., above 30 Hz), have been consistently reported among the myriad neurophysiological abnormalities seen in schizophrenia (8, 9, 11–17), and are associated with multiple cognitive deficits in patients (18). In contrast to the widely studied stimulus- or task-evoked gamma oscillations, spontaneous oscillatory abnormalities in schizophrenia, particularly in the gamma band, have been relatively less studied.

Resting-state EEG does not require behavioral responses to stimuli or cognitive tasks for elicitation and is already widely used as part of routine neurologic and psychiatric assessments (19). Spontaneous oscillations arise from the synchronous firing of neurons in distributed neuronal networks and are characterized at broadband frequency ranges detectable *via* scalp sensors. Such oscillations can also be characterized *via* the flow of spectral information among their calculated neural sources. Identification of the primary contributing neural sources as well as the dynamic interactions among sources of spontaneous EEG activity may elucidate fundamental pathophysiologic abnormalities associated with the illness which may ultimately yield clinically relevant applications (biomarkers of illness, risk of illness, or sensitivity to therapeutic interventions).

A recent review article reported that schizophrenia patients showed increases in the canonical theta, alpha, and beta bands, but with no difference in the delta band activity in scalp level responses (20). Despite enthusiasm for measures of gamma band phase locking and synchronization to steady-state stimulation (8, 21), resting state gamma band activity has not been commonly studied. Moreover, while such scalp-level responses have been extensively described, the spatial information of neural network dynamics underlying frequency-band specific resting-state EEG activity in schizophrenia patients is largely unknown. To our knowledge, only one paper, by Andreou et al. (22) reported increased resting-state gamma-band functional connectivity across the Rolandic operculum, a region that includes superior temporal and inferior frontal gyri, in schizophrenia patients.

In this study, a novel multivector autoregressive modeling method was developed and applied to assess the effective connectivity of resting-state EEG activity among cortical sources in schizophrenia patients and healthy comparison subjects. This data-driven approach enables an analysis of cortical network dynamics with directed information flow [e.g., Granger causality (23); increased or decreased EEG phase coherence between two cortical regions] using a correlation with a time delay. We hypothesized that patients with schizophrenia would show abnormal increased frequency-specific oscillations (e.g., gamma-band activity) across frontotemporal cortical networks. Furthermore, we aimed to characterize the networks associated with other frequency bands in schizophrenia patients and healthy comparison subjects.

## Materials and Methods

### Subjects

EEG data from *n* = 147 healthy comparison subjects and *n* = 159 schizophrenia patients was processed. Recordings from *n* = 2 healthy comparison subjects and *n* = 5 schizophrenia patients were dropped in the quality control step in the pre-processing of EEG. In the sample of *n* = 145 healthy comparison subjects and *n* = 154 schizophrenia patients, age and sex were significantly different between the groups. Therefore, we removed the subjects of extreme value of age and sex, and used a final sample of *n* = 126 healthy comparison subjects and *n* = 139 schizophrenia patients in the effective connectivity analysis (Supplementary Method 1, Supplementary Table 1). Resting-state spectral characteristics assessed at a single principal component analysis (PCA)-based composite scalp sensor level were previously reported (24). Antipsychotics, anxiolytics, and anticholinergics were prescribed for 125, 27, and 42 schizophrenia patients, respectively. Since anxiolytics and anticholinergic medications are known to have potential impacts on resting state scalp EEG (25, 26), separate analyses of schizophrenia patients who did not have either anxiolytics nor anticholinergics (*N* = 80) were also conducted. Written informed consent was obtained from each subject. The Institutional Review Board of University of California San Diego approved all experimental procedures (071831, 170147).

### Electroencephalography Recording and Pre-processing

Participants sat in a comfortable chair in a quiet room and were instructed to relax and with their eyes open. Subjects were closely monitored *via* a one-way mirror throughout this brief 5 min session. The recording could be paused if subjects appeared to be drowsy either by direct observation or as indicated in their EEG/EOG recordings. The recording would then be resumed after the subject was reminded to keep their eyes open.

EEG was continuously digitized at a rate of 1,000 Hz (nose reference, forehead ground) using a 40-channel Neuroscan system (Neuroscan Laboratories, El Paso, Texas). The electrode montage was based on standard positions in the International 10–5 electrode system (27) fit to the Montreal Neurological Institute template head used in EEGLAB (28). The system acquisition band pass was 0.5–100 Hz. Offline, EEG data were imported to EEGLAB 14.1.2 (29) running under Matlab 2017b (The MathWorks, Natick, MA). Data were high-pass filtered [finite impulse response (FIR), Hamming window, cutoff frequency 0.5 Hz, transition bandwidth 0.5]. EEGLAB plugin *clean_rawdata()* including artifact subspace reconstruction (ASR) was applied to reduce high-amplitude artifacts (30–35). The parameters used were: flat line removal, 10 s; electrode correlation, 0.7; ASR, 20; window rejection, 0.5. Mean channel rejection rate was 4.2 % [standard deviation (SD) 2.3, range 0–15.8]. Mean data rejection rate was 2.0% (SD 3.5, range 0–22.4). The rejected channels were interpolated using EEGLAB's spline interpolation function. Data were re-referenced to average. Adaptive mixture independent component analysis (ICA) was applied to the pre-processed scalp recording data to obtain temporally maximally independent components (ICs).

### Source Localization Using an Equivalent Current Dipole Model

The values in the column of the mixing matrix derived from ICA were mapped on to the scalp electrodes to obtain IC scalp topography, which represents scalp projection of ICA-derived effective EEG sources inside the brain (36). A previous study showed that this scalp topography is modeled well by an equivalent current dipole model, and in fact the “dipolarity” of IC scalp topography correlates with the mutual information reduced by ICA (37). Thus, even though ICA is agnostic on spatial information (electrode locations, electric forward model of the brain, or spatial information about location of the EEG generators), minimizing the mutual information in the decomposed signals naturally achieves a physiologically valid dipolar spatial projection pattern. These findings are often taken as evidence of physiological validity of ICA when applied to scalp-recorded EEG data (independence-dipolarity identity). This estimation of equivalent current dipoles was performed using Fieldtrip functions (38). Two symmetrical dipoles were estimated for scalp topographies (39).

### Selection of Independent Components Representing EEG

To select brain ICs among all types of ICs, EEGLAB plugin *ICLabel()* was used (40). The inclusion criteria were (1) “brain” label probability > 0.7 and (2) residual variance i.e., var[(actual scalp topography) – (theoretical scalp projection from the fitted dipole)]/var(actual scalp topography) <0.15. Seven subjects were removed because they did not have minimum of 4 brain ICs. The mean number of ICs remained was 12.5 (SD 4.5, range 4–25). To ensure consistency across computations, recordings longer than 300 s were truncated to 300 s. Mean data length was 297.7 s (SD 8.9, range 202–300).

### Effective Connectivity Analyses

To calculate the grand-mean effective connectivity across ICs for each group, we applied EEGLAB plugin groupSIFT, which recently demonstrated successful application in other neuropsychiatric disorders (41). Renormalized partial directed coherence [RPDC (42)] was calculated across ICs (single window, logarithmically distributing 50 frequency bins from 2 to 55 Hz). This generated a connectivity matrix with the dimension of IC × IC for each participant. The grand-average optimum model order determined *via* the elbow detection method was 7.1 (SD 0.6) i.e. delayed effective connectivity up to about 64 ms was utilized. An autocorrelation function (ACF) test showed that probability for the residual to be white was 0.81 (SD 0.04). Data consistency (43) was 88.2 % (SD 4.3). The estimated equivalent dipole locations of the corresponding ICs were convolved with 3-D Gaussian kernel with 20 mm full width at half maximum (FWHM) to obtain probabilistic dipole density (truncated at 3 σ). The dipole density inside the brain space is segmented into anatomical regions defined by custom automated anatomical labeling [AAL (44)]; the original 88 anatomical regions in AAL were reduced to 76 by summarizing basal and deep limbic regions into two umbrella regions, upper and lower basal. The labels “upper basal” and “lower basal” were originally matched to ventral mid-cingulate, “mid-cingulate” as dorsal mid-cingulate, and “insula” as inferior frontal. The individual IC × IC connectivity matrix was thus mapped to a 76 × 76 connectivity matrix, on which RPDC was also mapped as a weighting factor to modulate pairwise dipole density to calculate graph edges. For both groups (healthy comparison subjects and schizophrenia patients), including a minimum of 70% of unique subjects was set to be an inclusion criterion for each graph node to be analyzed in the next stage. Also, for the group comparison (healthy comparison subjects and schizophrenia patients), 48/76 graph nodes showed overlap between the groups, which explained 82.3% of total dipole density, consistent with findings from Loo et al. (41). For the statistics of RPDC in the frequency domain, a weak family-wise error rate control was applied (45, 46). The brain graphs were visualized using BrainNet Viewer software (47).

## Results

**Figure 1:**
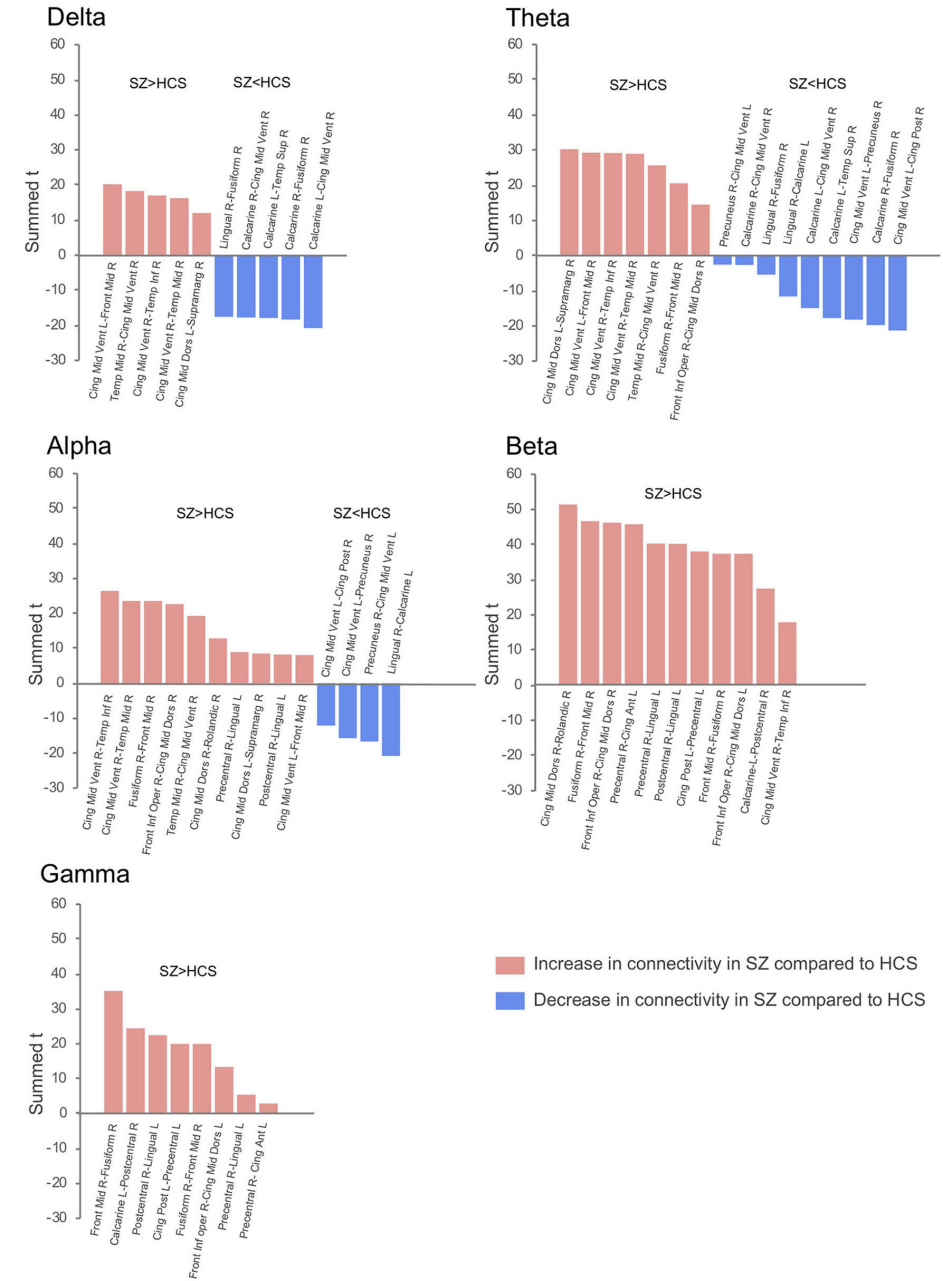
Difference of effective connectivity in each EEG band activity between healthy subjects (*N* = 126) and schizophrenia patients (*N* = 139). SZ, schizophrenia; HCS, healthy comparison subject; L, left; R, right; Front Mid, middle frontal; Front Inf Oper, opercular part of inferior frontal; Cing Ant, anterior cingulate; Cing Mid Dors, dorsal middle cingulate; Cing Mid Vent, ventral middle cingulate; Cing Post, posterior cingulate; Temp Sup, superior temporal; Temp Mid, middle temporal; Temp Inf, inferior temporal; Rolandic, Rolandic operculum; Supramarg, Supramarginal.

**Figure 2:**
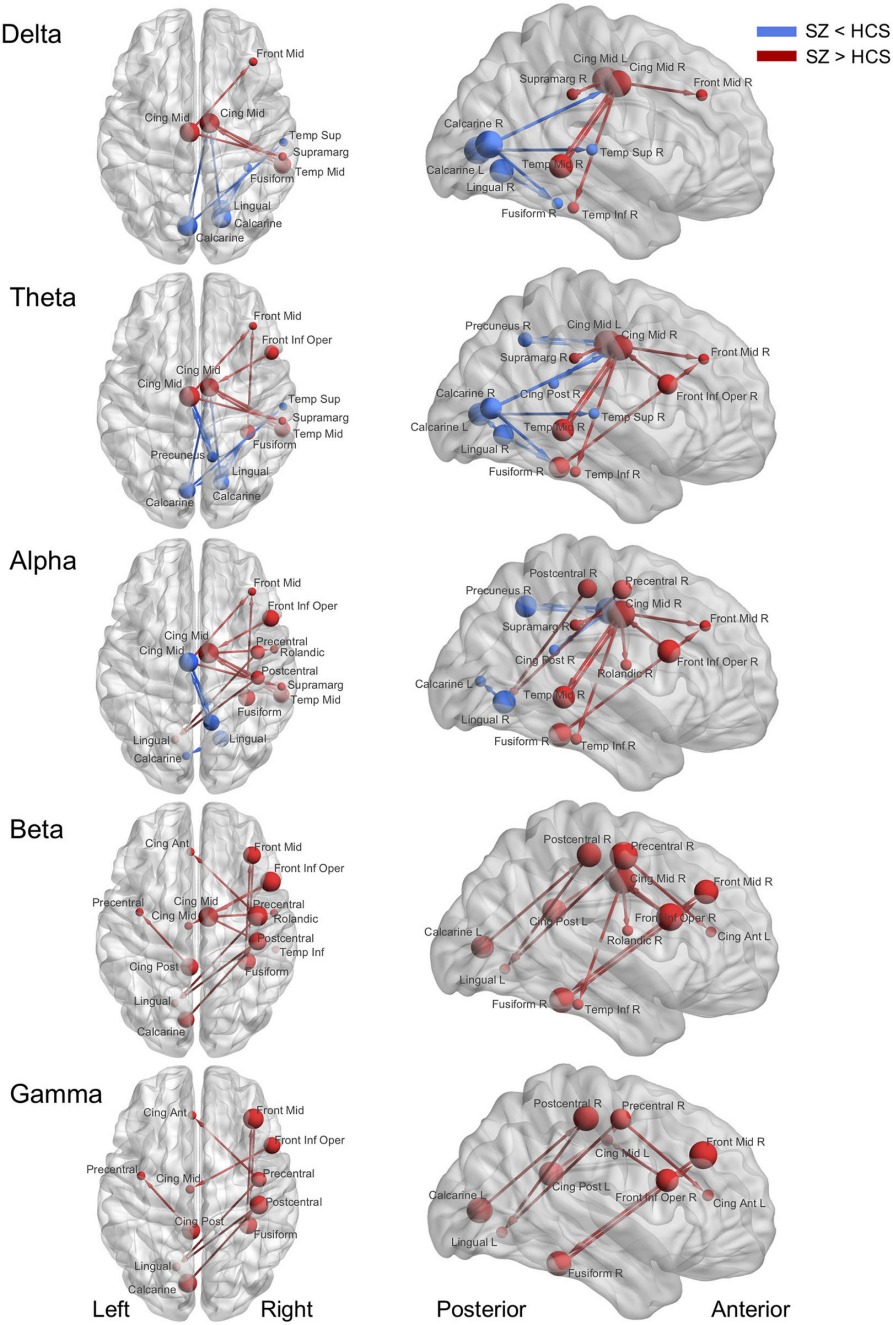
Abnormal effective connectivity of resting-state EEG activity in schizophrenia patients. Red arrow indicates high effective connectivity and blue arrow indicates low connectivity in schizophrenia patients relative to healthy comparison subjects. Sphere size indicate amount of total outflow in each node. SZ, schizophrenia; HCS, healthy comparison subject; Front Mid, middle frontal; Front Inf Oper, opercular part of inferior frontal; Cing Ant, anterior cingulate; Cing Mid Dors, dorsal middle cingulate; Cing Mid Vent, ventral middle cingulate; Cing Post, posterior cingulate; Temp Sup, superior temporal; Temp Mid, middle temporal; Temp Inf, inferior temporal; Rolandic, Rolandic operculum; Supramarg, Supramarginal.

**Figure 3:**
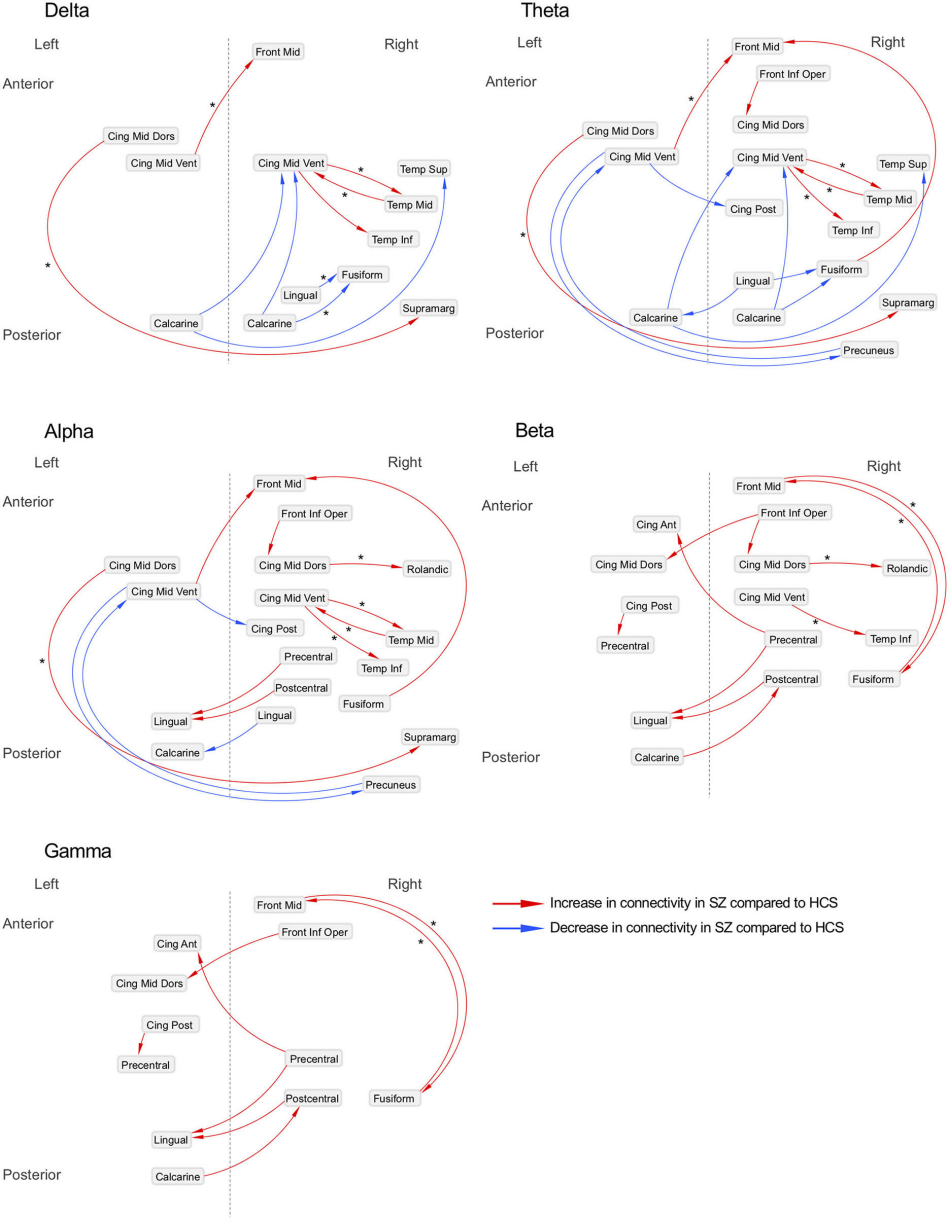
Neural networks underlying resting-state EEG activity in schizophrenia patients. Difference of effective connectivity between healthy subjects (*N* = 126) and schizophrenia patients (*N* = 139) is shown; Asterisks (*) indicate the increased or decreased information flows observed in schizophrenia patients who did not have either anxiolytics nor anticholinergics (*N* = 80) compared to healthy subjects (*N* = 126). SZ, schizophrenia; HCS, healthy comparison subject; Front Mid, middle frontal; Front Inf Oper, opercular part of inferior frontal; Cing Ant, anterior cingulate; Cing Mid Dors, dorsal middle cingulate; Cing Mid Vent, ventral middle cingulate; Cing Post, posterior cingulate; Temp Sup, superior temporal; Temp Mid, middle temporal; Temp Inf, inferior temporal; Rolandic, Rolandic operculum; Supramarg, Supramarginal.

The connectivity matrix that represents the group-difference [healthy subjects (*N* = 126) and schizophrenia patients (*N* = 139)] of each EEG band activity [a pre-defined *p* < 0.0001, corrected; two-tailed (48)] is shown in Supplementary Figure 1. The results revealed 10 graph edges (effective connectivity, i.e., increased or decreased EEG phase coherence between two cortical regions) for delta band (1–4 Hz), 16 for theta band (4–8 Hz), 14 for alpha band (8–14 Hz), 11 for beta band (14–30 Hz) and 8 for gamma band (30–50 Hz) activity (Figures 1–3). The connectivity results of healthy comparison subjects and schizophrenia patients are separately shown in Supplementary Figure 2.

The matrix of the group-difference [healthy subjects (*N* = 126) and schizophrenia patients who were not treated with anxiolytics or anticholinergics (*N* = 80)] of each EEG band activity (*p* < 0.0001, corrected; two-tailed) is also shown in Supplementary Figure 3. We revealed 10 graph edges for delta band, 14 for theta band, 17 for alpha band, 17 for beta band and 6 for gamma band activity (Supplementary Figures 4, 5).

### Delta Band Activity (1–4 Hz)

Decreased effective connectivity from a region near the calcarine sulcus to the fusiform, temporal and middle cingulate gyri was detected in delta band in schizophrenia patients compared to healthy subjects (Figures 1–3). A bidirectional increased interaction between the right middle temporal gyrus and the right middle cingulate gyrus was also observed. These connectivities were more prominent in the right hemisphere.

### Theta Band Activity (4–8 Hz)

Theta band effective connectivity demonstrated a similar right-sided asymmetry centered on the temporal and middle cingulate gyri in schizophrenia relative to healthy subjects (Figures 1–3). Decreased effective connectivity from a region near the calcarine sulcus to the fusiform, temporal and middle cingulate gyri was also detected in theta band activity in schizophrenia patients compared to healthy subjects. The bidirectional increased interaction between the right middle cingulate gyrus and right middle temporal gyrus was also seen in theta band activity in schizophrenia patients relative to healthy subjects. Increased effective connectivity from the right fusiform gyrus to the right middle frontal gyrus was seen in schizophrenia relative to healthy subjects.

### Alpha Band Activity (8–14 Hz)

The overall pattern of alpha connectivity is similar with those observed in theta band activity (Figures 1–3). Increased effective connectivity from the right middle cingulate gyrus to the Rolandic operculum (a region that includes auditory cortex and spans Broadmann areas 41 and 42) was detected in schizophrenia relative to healthy subjects. Increased effective connectivity from the right fusiform gyrus to the right middle frontal gyrus and the bidirectional increased interaction between the right middle cingulate gyrus and the right middle temporal gyrus were also seen in schizophrenia relative to healthy subjects.

### Beta Band Activity (14–30 Hz)

Abnormal patterns of connectivity were observed among temporal, middle cingulate and occipital regions. These networks overlapped across beta and alpha band activity in schizophrenia patients compared to healthy subjects (Figures 1–3). Increased effective connectivity from the right middle cingulate gyrus to the Rolandic operculum was also seen in schizophrenia relative to healthy subjects. Increased bidirectional information flows between the right middle frontal gyrus and the right fusiform gyrus were detected in schizophrenia patients compared to healthy subjects.

### Gamma Band Activity (30–50 Hz)

The increased bidirectional information flows between the right middle frontal gyrus and the right fusiform gyrus were also detected in gamma band activity in schizophrenia patients compared to healthy subjects (Figures 1–3). Although the abnormal neural network was overlapped across gamma and beta band activity in patients compared to healthy subjects, the overall structure was simpler and more localized for gamma vs. beta band activity. This relatively simpler structure for gamma band suggests that higher frequency abnormal networks in schizophrenia compared to healthy subjects consisted of more independent local networks that therefore did not connect with other regions.

## Discussion

**Figure 4:**
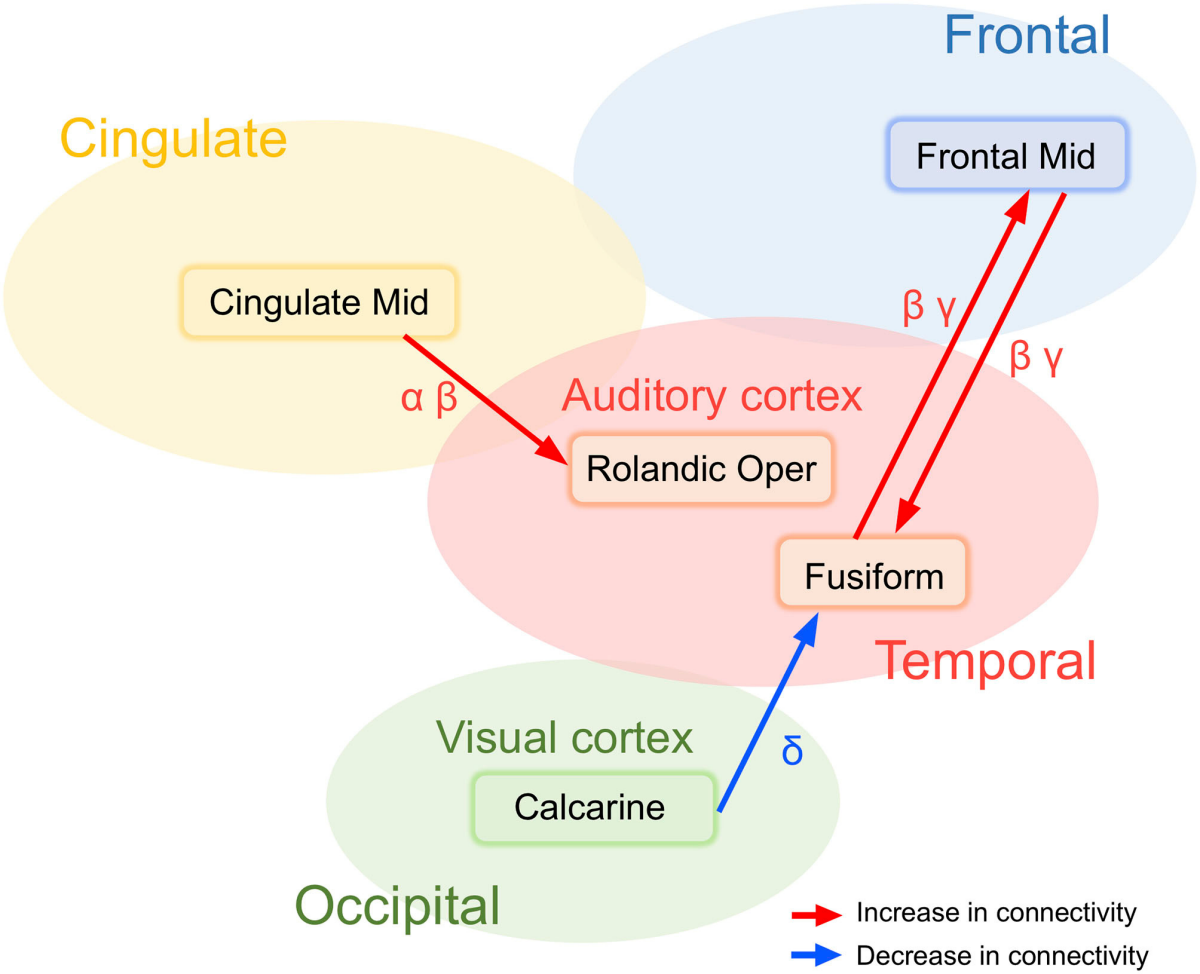
Two major abnormal networks associated with either visual and auditory information processing detected in schizophrenia patients at rest.

Schizophrenia patients showed broad and widespread hyper-connectivity of cortical networks underlying resting-state EEG activity. Specifically, the following findings were detected; (1) decreased information flows from a region near the right calcarine sulcus to the right fusiform gyrus in delta band activity, and bidirectionally increased interactions between the right fusiform gyrus and the right middle frontal gyrus in beta and gamma band activity (i.e., “visual network”; Figure 4); (2) Increased information flow from the right middle cingulate gyrus to the Rolandic operculum across alpha and beta bands in schizophrenia patients compared to healthy subjects (i.e., “auditory network”; Figure 4); With few minor exceptions, these results were largely confirmed in a subgroup of schizophrenia patients who were not on anxiolytics or anticholinergics.

The present results replicate abnormal effective connectivity between frontotemporal regions in schizophrenia patients (22). Increased functional connectivity of alpha band activity at the superior parietal and the occipital lobe area at scalp levels of EEG in schizophrenia patients (*N* = 28) were previously reported by Liu et al. (49); we successfully replicate and extended the results showing the involvement of frontotemporal regions. We also previously reported that abnormal spontaneous gamma band activity measured *via* a spatial PCA of scalp channel data was associated with verbal memory performance (24). Although the PCA method used in the prior study provides a data-driven approach for characterizing macroscale/global oscillatory effects at the scalp, the neural interactions among sources were not assessed. The current results suggest that the spontaneous global gamma band abnormalities observed in schizophrenia patients at the scalp level appear to be generated by interactions between prefrontal and temporal regions.

A previous study by Andreou reported increased theta-band resting-state connectivity across midline, sensorimotor, orbitofrontal regions and the left temporoparietal junction in schizophrenia patients (*N* = 19) (50), consistent with our findings of right dominant increased effective connectivity among the temporal and middle cingulate gyri in broad band EEG activity including theta band activity. Inconsistencies in the laterality of effects, however, may be due to the difference of age or clinical severity. For example, the mean age of schizophrenia patients in the Andreou et al. study (50) was 23.5 vs. 44.6 years in the current study. Despite this difference in theta, our finding of increased alpha connectivity at the right temporal region at scalp levels of EEG in schizophrenia patients is fully consistent with the findings of Liu et al. (49).

Of note, despite the temporal differences between neural activity detected by very low frequency blood oxygenation-dependent (BOLD) hemodynamic responses and EEG, our resting-state EEG connectivity findings are also fully compatible with functional magnetic resonance imaging study (fMRI) findings of default mode network abnormalities in schizophrenia patients (51). The present findings of greater connectivity between the middle frontal, anterior cingulate and middle temporal gyri regions, is consistent with greater default mode network activation in schizophrenia (52).

### Networks Centered at the Visual Cortex

The effective connectivity networks centered in the calcarine sulcus and the fusiform gyrus in broad band EEG activity were unexpected. The calcarine sulcus is a deep fissure that starts in the temporal lobe that continues into the occipital lobe with the primary visual cortex (V1) centered in this region. The fusiform gyrus is large region in the inferior temporal cortex that also has a functional role in visual information processing (53), including object and face recognition, and the recognition of facial expressions (Figure 4). Indeed, despite these unexpected associations, results are consistent with Morita et al. (54) findings of associated eye movement impairments with gray matter cortical thickness in schizophrenia patients in the middle frontal and fusiform gyri and the lateral occipital cortex. Although speculative, patients with schizophrenia may show abnormal excessive simultaneous activation of various perception-related brain regions, which may ultimately contribute to clinical symptoms such as hallucinations, aberrant salience, and delusions.

### Networks Centered at the Auditory Cortex

Increased information flows were detected in schizophrenia patients from the right middle cingulate gyrus converging on the right Rolandic operculum (Figure 4). In the current analysis, primary auditory cortex is located in the region labeled the Rolandic operculum. Previous studies have demonstrated that deficits in early auditory information processing in schizophrenia patients as indexed by mismatch negativity (48, 55) and gamma-band auditory steady-state responses (9, 56, 57) are supported by distributed networks where the genesis of the responses are detected in the superior temporal gyrus (a region that includes auditory cortex) which subsequently propagate across other temporal and frontal brain regions. The present results suggest that resting state abnormalities in schizophrenia patients are present across multiple frequency bands and over relatively large spatial networks. Measures of network connectivity from cingulate gyrus to the auditory cortex may be therefore account for multiple neurophysiologic biomarkers and show promise as a future candidate biomarker of abnormalities in schizophrenia patients.

### Limitations

Results of this study should be considered in the context of several limitations. First, this is a cross-sectional cohort study of a heterogeneous sample of schizophrenia patients, the majority of whom were prescribed complex medication regimens. While comparisons of patients prescribed vs. not-prescribed medications that are known to impact resting state scalp responses (i.e., anxiolytics or anticholinergics) and healthy subjects showed similar patterns of results, it is possible that other medications including antipsychotics or symptoms may contribute to the observation of abnormal network dynamics. Carefully controlled prospective randomized controlled trials are needed to disentangle medication effects. Despite efforts were made to obtain medical/prescription records for all subjects, self- and informant reports of medication compliance, ultimately medication compliance could not be confirmed for the majority of patients in this study. As such, more rigorous analyses of medication doses and connectivity analyses were not pursued. Second, only 40 EEG channels were used for the analyses in this study. Future studies may benefit from the use of high-density EEG recordings with at least 64 channels (58), individual MRI data, and digitized scalp sensor locations rather than template head models and reliance on standardized electrode locations for potentially improved accuracy of source dynamics. Third, while we believe that elaboration of neural system dynamics reported here will be broadly applicable to multiple neuropsychiatric disorders, we acknowledge the possibility that results from schizophrenia patients with an established illness may not generalize to other populations like clinical high risk or first episode psychosis. Nonetheless, given improvements in medical care and life expectancy, patients with more chronic schizophrenia are likely to represent an increasing proportion of the total schizophrenia population; characterization of abnormal network dynamics among real-world patients *via* data-driven approaches for assessing network dynamics may ultimately be useful for application as biomarkers the development of therapeutics for this largely underserved population.

## Conclusions

Results of this study provide evidence that abnormal resting-state EEG oscillations are driven by patterns of hyper-connectivity across multiple frequency bands and a distributed network of the frontal, temporal and occipital brain regions that are involved in visual and auditory information processing in schizophrenia patients. Future studies of the neural mechanisms underlying the networks detected in this study, in both future clinical and animal studies, are needed to clarify the pathophysiology of neuropsychiatric and neurological diseases in support of the development of novel therapeutic interventions.

## Data Availability Statement

The datasets generated for this article are not readily available to third parties. Requests to access the datasets should be directed to Gregory A. Light, glight@health.ucsd.edu.

## Ethics Statement

The studies involving human participants were reviewed and approved by Institutional Review Board of University of California San Diego. The patients/participants provided their written informed consent to participate in this study.

## Author Contributions

JS and GL collected the data. DK and MM analyzed the data. MM wrote the Matlab code. DK, MM, YJ, JM, KT-K, DB, and GL interpreted the results. DK, MM, and GL designed the study. GL supervised all aspects of collection, analysis, and interpretation of the data. DK, MM, and GL wrote original manuscript. YJ, JM, KT-K, JS, and DB reviewed and edited the manuscript. All authors contributed to and approved the final manuscript.

## Conflict of Interest

The authors declare that the research was conducted in the absence of any commercial or financial relationships that could be construed as a potential conflict of interest.

## References

[1] Joliot M Ribary U Llinas R. Human oscillatory brain activity near 40 Hz coexists with cognitive temporal binding. Proc Natl Acad Sci U S A. (1994) 91:11748–51. 10.1073/pnas.91.24.11748 7972135 PMC45309

[2] Traub RD Whittington MA Stanford IM Jefferys JG. A mechanism for generation of long-range synchronous fast oscillations in the cortex. Nature. (1996) 383:621–4. 10.1038/383621a0 8857537

[3] Miltner WH Braun C Arnold M Witte H Taub E. Coherence of gamma-band EEG activity as a basis for associative learning. Nature. (1999) 397:434–6. 10.1038/17126 9989409

[4] Rodriguez E George N Lachaux JP Martinerie J Renault B Varela FJ. Perception's shadow: long-distance synchronization of human brain activity. Nature. (1999) 397:430–3. 10.1038/17120 9989408

[5] Hagoort P Hald L Bastiaansen M Petersson KM. Integration of word meaning and world knowledge in language comprehension. Science. (2004) 304:438–41. 10.1126/science.1095455 15031438

[6] Spellman T Rigotti M Ahmari SE Fusi S Gogos JA Gordon JA. Hippocampal-prefrontal input supports spatial encoding in working memory. Nature. (2015) 522:309–14. 10.1038/nature14445 26053122 PMC4505751

[7] Galuske RAW Munk MHJ Singer W. Relation between gamma oscillations and neuronal plasticity in the visual cortex. Proc Natl Acad Sci U S A. (2019) 116:23317–25. 10.1073/pnas.1901277116 31659040 PMC6859324

[8] Uhlhaas PJ Singer W. Abnormal neural oscillations and synchrony in schizophrenia. Nat Rev Neurosci. (2010) 11:100–13. 10.1038/nrn2774 20087360

[9] Hirano Y Oribe N Kanba S Onitsuka T Nestor PG Spencer KM. Spontaneous gamma activity in schizophrenia. JAMA Psychiatry. (2015) 72:813–21. 10.1001/jamapsychiatry.2014.2642 25587799 PMC4768724

[10] Sun Y Farzan F Barr MS Kirihara K Fitzgerald PB Light GA. Gamma oscillations in schizophrenia: mechanisms and clinical significance. Brain Res. (2011) 1413:98–114. 10.1016/j.brainres.2011.06.065 21840506

[11] Molina JL Voytek B Thomas ML Joshi YB Bhakta SG Talledo JA. Memantine effects on electroencephalographic measures of putative excitatory/inhibitory balance in schizophrenia. Biol Psychiatry Cogn Neurosci Neuroimaging. (2020) 5:562–8. 10.1016/j.bpsc.2020.02.004 32340927 PMC7286803

[12] Thune H Recasens M Uhlhaas PJ. The 40-Hz auditory steady-state response in patients with schizophrenia: a meta-analysis. JAMA Psychiatry. (2016) 73:1145–53. 10.1001/jamapsychiatry.2016.2619 27732692

[13] Spencer KM Salisbury DF Shenton ME McCarley RW. Gamma-band auditory steady-state responses are impaired in first episode psychosis. Biol Psychiatry. (2008) 64:369–75. 10.1016/j.biopsych.2008.02.021 18400208 PMC2579257

[14] Tada M Kirihara K Koshiyama D Fujioka M Usui K Uka T. Gamma-band auditory steady-state response as a neurophysiological marker for excitation and inhibition balance: a review for understanding schizophrenia and other neuropsychiatric disorders. Clin EEG Neurosci. (2019) 51:234–43. 10.1177/1550059419868872 31402699

[15] Koshiyama D Kirihara K Tada M Nagai T Fujioka M Ichikawa E. Auditory gamma oscillations predict global symptomatic outcome in the early stages of psychosis: a longitudinal investigation. Clin Neurophysiol. (2018) 129:2268–75. 10.1016/j.clinph.2018.08.007 30216911

[16] Koshiyama D Kirihara K Tada M Nagai T Fujioka M Ichikawa E. Electrophysiological evidence for abnormal glutamate-GABA association following psychosis onset. Transl Psychiatry. (2018) 8:211. 10.1038/s41398-018-0261-0 30297786 PMC6175929

[17] Koshiyama D Kirihara K Tada M Nagai T Fujioka M Usui K. Gamma-band auditory steady-state response is associated with plasma levels of d-serine in schizophrenia: an exploratory study. Schizophr Res. (2019) 208:467–9. 10.1016/j.schres.2019.02.012 30819595

[18] Senkowski D Gallinat J. Dysfunctional prefrontal gamma-band oscillations reflect working memory and other cognitive deficits in schizophrenia. Biol Psychiatry. (2015) 77:1010–9. 10.1016/j.biopsych.2015.02.034 25847179

[19] Feyissa AM Tatum WO. Adult EEG. Handb Clin Neurol. (2019) 160:103–24. 10.1016/B978-0-444-64032-1.00007-2 31277842

[20] Newson JJ Thiagarajan TC. EEG frequency bands in psychiatric disorders: a review of resting state studies. Front Hum Neurosci. (2018) 12:521. 10.3389/fnhum.2018.00521 30687041 PMC6333694

[21] Uhlhaas PJ Haenschel C Nikolic D Singer W. The role of oscillations and synchrony in cortical networks and their putative relevance for the pathophysiology of schizophrenia. Schizophr Bull. (2008) 34:927–43. 10.1093/schbul/sbn062 18562344 PMC2632472

[22] Andreou C Nolte G Leicht G Polomac N Hanganu-Opatz IL Lambert M. Increased resting-state gamma-band connectivity in first-episode schizophrenia. Schizophr Bull. (2015) 41:930–9. 10.1093/schbul/sbu121 25170031 PMC4466170

[23] Granger CWJ Investigating causal relations by econometric models and cross-spectral methods. Econometrica. (1969) 37:424–38. 10.2307/1912791

[24] Tanaka-Koshiyama K Koshiyama D Miyakoshi M Joshi YB Molina JL Sprock J. Abnormal spontaneous gamma power is associated with underlying verbal learning and memory dysfunction in schizophrenia. Front Psychiatry. (2020) 11:832. 10.3389/fpsyt.2020.00832 33110410 PMC7488980

[25] Buchsbaum MS Hazlett E Sicotte N Stein M Wu J Zetin M. Topographic EEG changes with benzodiazepine administration in generalized anxiety disorder. Biol Psychiatry. (1985) 20:832–42. 10.1016/0006-3223(85)90208-2 2862924

[26] Sloan EP Fenton GW Standage KP. Anticholinergic drug effects on quantitative electroencephalogram, visual evoked potential, and verbal memory. Biol Psychiatry. (1992) 31:600–6. 10.1016/0006-3223(92)90246-V 1581439

[27] Oostenveld R Praamstra P. The five percent electrode system for high-resolution EEG and ERP measurements. Clin Neurophysiol. (2001) 112:713–9. 10.1016/S1388-2457(00)00527-7 11275545

[28] Collins DL Neelin P Peters TM Evans AC. Automatic 3D intersubject registration of MR volumetric data in standardized Talairach space. J Comput Assist Tomogr. (1994) 18:192–205. 10.1097/00004728-199403000-00005 8126267

[29] Delorme A Makeig S. EEGLAB: an open source toolbox for analysis of single-trial EEG dynamics including independent component analysis. J Neurosci Methods. (2004) 134:9–21. 10.1016/j.jneumeth.2003.10.009 15102499

[30] Blum S Jacobsen NSJ Bleichner MG Debener S. A riemannian modification of artifact subspace reconstruction for EEG artifact handling. Front Hum Neurosci. (2019) 13:141. 10.3389/fnhum.2019.00141 31105543 PMC6499032

[31] Chang CY Hsu SH Pion-Tonachini L Jung TP. Evaluation of artifact subspace reconstruction for automatic artifact components removal in multi-channel EEG recordings. IEEE Trans Biomed Eng. (2020) 67:1114–21. 10.1109/TBME.2019.2930186 31329105

[32] Chang CY Hsu SH Pion-Tonachini L Jung TP. Evaluation of artifact subspace reconstruction for automatic EEG artifact removal. Conf Proc IEEE Eng Med Biol Soc. (2018) 2018:1242–5. 10.1109/EMBC.2018.8512547 30440615

[33] Gabard-Durnam LJ Mendez Leal AS Wilkinson CL Levin AR. The Harvard automated processing pipeline for electroencephalography (HAPPE): standardized processing software for developmental and high-artifact data. Front Neurosci. (2018) 12:97. 10.3389/fnins.2018.00097 29535597 PMC5835235

[34] Kothe CA Makeig S. BCILAB: a platform for brain-computer interface development. J Neural Eng. (2013) 10:056014. 10.1088/1741-2560/10/5/056014 23985960

[35] Mullen TR Kothe CA Chi YM Ojeda A Kerth T Makeig S. Real-time neuroimaging and cognitive monitoring using wearable dry EEG. IEEE Trans Biomed Eng. (2015) 62:2553–67. 10.1109/TBME.2015.2481482 26415149 PMC4710679

[36] Onton J Makeig S. Information-based modeling of event-related brain dynamics. Prog Brain Res. (2006) 159:99–120. 10.1016/S0079-6123(06)59007-7 17071226

[37] Delorme A Palmer J Onton J Oostenveld R Makeig S. Independent EEG sources are dipolar. PLoS ONE. (2012) 7:e30135. 10.1371/journal.pone.0030135 22355308 PMC3280242

[38] Oostenveld R Fries P Maris E Schoffelen JM FieldTrip: open source software for advanced analysis of MEG, EEG, and invasive electrophysiological data. Comput Intell Neurosci. (2011) 2011:156869 10.1155/2011/156869 21253357 PMC3021840

[39] Piazza C Miyakoshi M Akalin-Acar Z Cantiani C Reni G Bianchi AM An automated function for identifying eeg independent components representing bilateral source activity. In: XIV Mediterranean Conference on Medical and Biological Engineering and Computing 2016. Paphos (2016). p. 105–9.

[40] Pion-Tonachini L Kreutz-Delgado K Makeig S. ICLabel: an automated electroencephalographic independent component classifier, dataset, and website. Neuroimage. (2019) 198:181–97. 10.1016/j.neuroimage.2019.05.026 31103785 PMC6592775

[41] Loo SK Miyakoshi M Tung K Lloyd E Salgari G Dillon A. Neural activation and connectivity during cued eye blinks in Chronic Tic disorders. Neuroimage Clin. (2019) 24:101956. 10.1016/j.nicl.2019.101956 31382238 PMC6698693

[42] Schelter B Timmer J Eichler M. Assessing the strength of directed influences among neural signals using renormalized partial directed coherence. J Neurosci Methods. (2009) 179:121–30. 10.1016/j.jneumeth.2009.01.006 19428518

[43] Ding M Bressler SL Yang W Liang H. Short-window spectral analysis of cortical event-related potentials by adaptive multivariate autoregressive modeling: data preprocessing, model validation, and variability assessment. Biol Cybern. (2000) 83:35–45. 10.1007/s004229900137 10933236

[44] Tzourio-Mazoyer N Landeau B Papathanassiou D Crivello F Etard O Delcroix N. Automated anatomical labeling of activations in SPM using a macroscopic anatomical parcellation of the MNI MRI single-subject brain. Neuroimage. (2002) 15:273–89. 10.1006/nimg.2001.0978 11771995

[45] Nichols T Hayasaka S. Controlling the familywise error rate in functional neuroimaging: a comparative review. Stat Methods Med Res. (2003) 12:419–46. 10.1191/0962280203sm341ra 14599004

[46] Groppe DM Urbach TP Kutas M. Mass univariate analysis of event-related brain potentials/fields I: a critical tutorial review. Psychophysiology. (2011) 48:1711–25. 10.1111/j.1469-8986.2011.01273.x 21895683 PMC4060794

[47] Xia M Wang J He Y. BrainNet viewer: a network visualization tool for human brain connectomics. PLoS ONE. (2013) 8:e68910. 10.1371/journal.pone.0068910 23861951 PMC3701683

[48] Koshiyama D Miyakoshi M Joshi YB Molina JL Tanaka-Koshiyama K Sprock J. Abnormal effective connectivity underlying auditory mismatch negativity impairments in schizophrenia. Biol Psychiary Cogn Neurosci Neuroimaging. (2020) 5:1028–39. 32830097 10.1016/j.bpsc.2020.05.011

[49] Liu T Zhang J Dong X Li Z Shi X Tong Y. Occipital alpha connectivity during resting-state electroencephalography in patients with ultra-high risk for psychosis and schizophrenia. Front Psychiatry. (2019) 10:553. 10.3389/fpsyt.2019.00553 31474882 PMC6706463

[50] Andreou C Leicht G Nolte G Polomac N Moritz S Karow A. Resting-state theta-band connectivity and verbal memory in schizophrenia and in the high-risk state. Schizophr Res. (2015) 161:299–307. 10.1016/j.schres.2014.12.018 25553979

[51] Buckner RL Andrews-Hanna JR Schacter DL. The brain's default network: anatomy, function, and relevance to disease. Ann N Y Acad Sci. (2008) 1124:1–38. 10.1196/annals.1440.011 18400922

[52] Garrity AG Pearlson GD McKiernan K Lloyd D Kiehl KA Calhoun VD. Aberrant “default mode” functional connectivity in schizophrenia. Am J Psychiatry. (2007) 164:450–7. 10.1176/ajp.2007.164.3.450 17329470

[53] Weiner KS Zilles K. The anatomical and functional specialization of the fusiform gyrus. Neuropsychologia. (2016) 83:48–62. 10.1016/j.neuropsychologia.2015.06.033 26119921 PMC4714959

[54] Morita K Miura K Fujimoto M Yamamori H Yasuda Y Kudo N. Eye-movement characteristics of schizophrenia and their association with cortical thickness. Psychiatry Clin Neurosci. (2019) 73:508–9. 10.1111/pcn.12865 31102322

[55] Salisbury DF Kuroki N Kasai K Shenton ME McCarley RW. Progressive and interrelated functional and structural evidence of post-onset brain reduction in schizophrenia. Arch Gen Psychiatry. (2007) 64:521–9. 10.1001/archpsyc.64.5.521 17485604 PMC2903200

[56] Koshiyama D Miyakoshi M Joshi YB Molina JL Tanaka-Koshiyama K Sprock J. A distributed frontotemporal network underlies gamma-band synchronization impairments in schizophrenia patients. Neuropsychopharmacology. (2020) 45:2198–206. 32829382 10.1038/s41386-020-00806-5 PMC7784692

[57] Hirano Y Oribe N Onitsuka T Kanba S Nestor PG Hosokawa T. Auditory cortex volume and gamma oscillation abnormalities in schizophrenia. Clin EEG Neurosci. (2020) 51:244–51. 10.1177/1550059420914201 32204613

[58] Light GA Swerdlow NR. Selection criteria for neurophysiologic biomarkers to accelerate the pace of CNS therapeutic development. Neuropsychopharmacology. (2020) 45:237–8. 10.1038/s41386-019-0519-0 31506611 PMC6879638

