# Supplementary material for: Neurophysiologic Characterization of Resting State Connectivity Abnormalities in Schizophrenia Patients
Source: Front Psychiatry. 2020 Nov 27;11:608154. doi: 10.3389/fpsyt.2020.608154 (PMC7729083; doi:10.3389/fpsyt.2020.608154)
Supplement: Supplementary file 1 [file Data_Sheet_1.pdf]

## Supplementary Information

# Neurophysiologic characterization of resting state connectivity abnormalities in schizophrenia patients

Daisuke Koshiyama, M.D., Ph.D.[1]; Makoto Miyakoshi, Ph.D.[2]; Kumiko Tanaka-Koshiyama, M.D.[1]; Yash B. Joshi, M.D., Ph.D.[1]; Juan L. Molina, M.D.[1]; Joyce Sprock, B.A.[1]; David L. Braff, M.D.[1]; Gregory A. Light, Ph.D.[1,3]

1. Department of Psychiatry, University of California San Diego, La Jolla, CA, USA
2. Swartz Center for Neural Computation, University of California San Diego, La Jolla, CA, USA
3. VISN-22 Mental Illness, Research, Education and Clinical Center (MIRECC), VA San Diego Healthcare System, San Diego, CA, USA

**TABLE OF CONTENTS**

**SUPPLEMENTARY METHOD**

**Supplementary Method 1** Subjects……………………………………….………………........2

**SUPPLEMENTARY TABLES**

**Supplementary Table 1** Demographic data of subjects……………………………….…......4

**SUPPLEMENTARY FIGURES**

**Supplementary Figure 1** Connectivity matrix of 76 × 76 anatomical region of interests (ROIs) for each band EEG activity…………………………………………………………….........5

**Supplementary Figure 2** Effective connectivity in each EEG band activity in healthy subjects (N=126) and schizophrenia patients (N=139)……………….……………...……..……...7

**Supplementary Figure 3** Connectivity matrix of 76 × 76 anatomical region of interests (ROIs) for each band EEG activity in healthy subjects (N=126) and schizophrenia patients who did not have either anxiolytics nor anticholinergics (N=80)……………………………...………9

**Supplementary Figure 4** Effective connectivity in each EEG band activity in healthy subjects (N=126) and schizophrenia patients who did not have either anxiolytics nor anticholinergics (N=80)…………………………………………………………………………………...11

**Supplementary Figure 5** Neural networks underlying resting-state EEG activity in schizophrenia patients who did not have either anxiolytics nor anticholinergics (N=80)……………………………………………………………………………...…13

## SUPPLEMENTARY METHOD

**Supplementary Method 1** Subjects

Patients were recruited from community residential facilities and via clinician referral. Diagnosis was confirmed using the Structured Clinical Interview for DSM-IV-TR. Healthy comparison subjects were recruited via internet advertisements. All subjects were excluded if they had any neurologic disease or damage, systemic medical illnesses that may compromise neurocognitive functioning (e.g. insulin dependent diabetes, heart disease), history of head injury with documented loss of consciousness, significant substance abuse during the prior six months, any history of detoxification/ hospitalization for substance abuse, physical handicaps that would interfere with assessment procedures, an inability to understand the consent processes and/or provide informed consent, or non-fluency in spoken or written English. In addition, all subjects received confidential urine toxicology screens for drugs of abuse and were excluded if the test was positive. Healthy comparison subjects who reported past treatment for a psychiatric disorder including hospitalization or electroconvulsive therapy, or having ever been treated with any psychoactive medications were excluded. In addition, those who reported having a first degree relative with schizophrenia or other psychotic disorders were excluded. Healthy comparison subjects were assessed with the SCID-Non Patient Edition (SCID-NP) (First et al. 1996a) and SCID-II (First et al. 1996b) for Axis II personality disorders. Those who met criteria for any past or current Axis I disorder were excluded as well any who met criteria for an Axis II Cluster A personality disorder.

Clinical symptoms in patients with schizophrenia were assessed with the Scale for the Assessment of Negative Symptoms (SANS; scores ranged from 0–25, with higher scores indicating severe symptom (Andreasen 1984a)) and the Scale for the Assessment of Positive Symptoms (SAPS; scores ranged from 0–20, with higher scores indicating severe symptom (Andreasen 1984b)). Functional outcome was measured using the Global Assessment of Functioning scale (GAF), a scale that evaluates the overall level of social adaptation from 0 to 100 scores with higher scores indicating a higher level of function.

### References

Andreasen NC. 1984a. The scale for the assessment of negative symptoms (SANS). Iowa CIty: University of Iowa.

Andreasen NC. 1984b. The scale for the assessment of positive symptoms (SAPS). Iowa CIty: University of Iowa.

First M, Spitzer R, Gibbon M, Williams J. 1996a. Structured Clinical Interview for DSM-IV Axis I Disorders -- Non-Patient Edition (SCID-I/NP, Version 2.0). New York: New York State Psychiatric Institute.

First M, Spitzer R, Gibbon M, Williams J, Benjamin L. 1996b. Structured Clinical Interview for DSM-IV Axis II Disorders (SCID-II, Version 2.0). New York: New York State Psychiatric Institute.

## SUPPLEMENTARY TABLES

**Supplementary Table 1** Demographic data of subjects

| | Healthy comparison subjects, N=126 | Schizophrenia patients, N=139 | Statistics |
|---|---|---|---|
| Gender (Male/Female) | 64/62 | 86/53 | $\chi^2$=3.3, *df* =1, $p$ = 0.07 |
| Age (year) | 42.0 (12.1) | 44.6 (10.0) | $t_{263}$= 1.9, $p$ = 0.06 |
| Duration of illness (year) | | 23.7 (11.2) | |
| SAPS | | 6.9 (4.2) | |
| SANS | | 16.9 (3.8) | |
| GAF | | 41.3 (4.4) | |

Legends: All values are shown as mean (standard deviation).

Abbreviations: SAPS, Scale for the Assessment of Positive Symptoms; SANS, Scale for the Assessment of Negative Symptoms; GAF, Global Assessment of Functioning.

## SUPPLEMENTARY FIGURES

**Supplementary Figure 1** Connectivity matrix of 76 × 76 anatomical region of interests (ROIs) for each band EEG activity

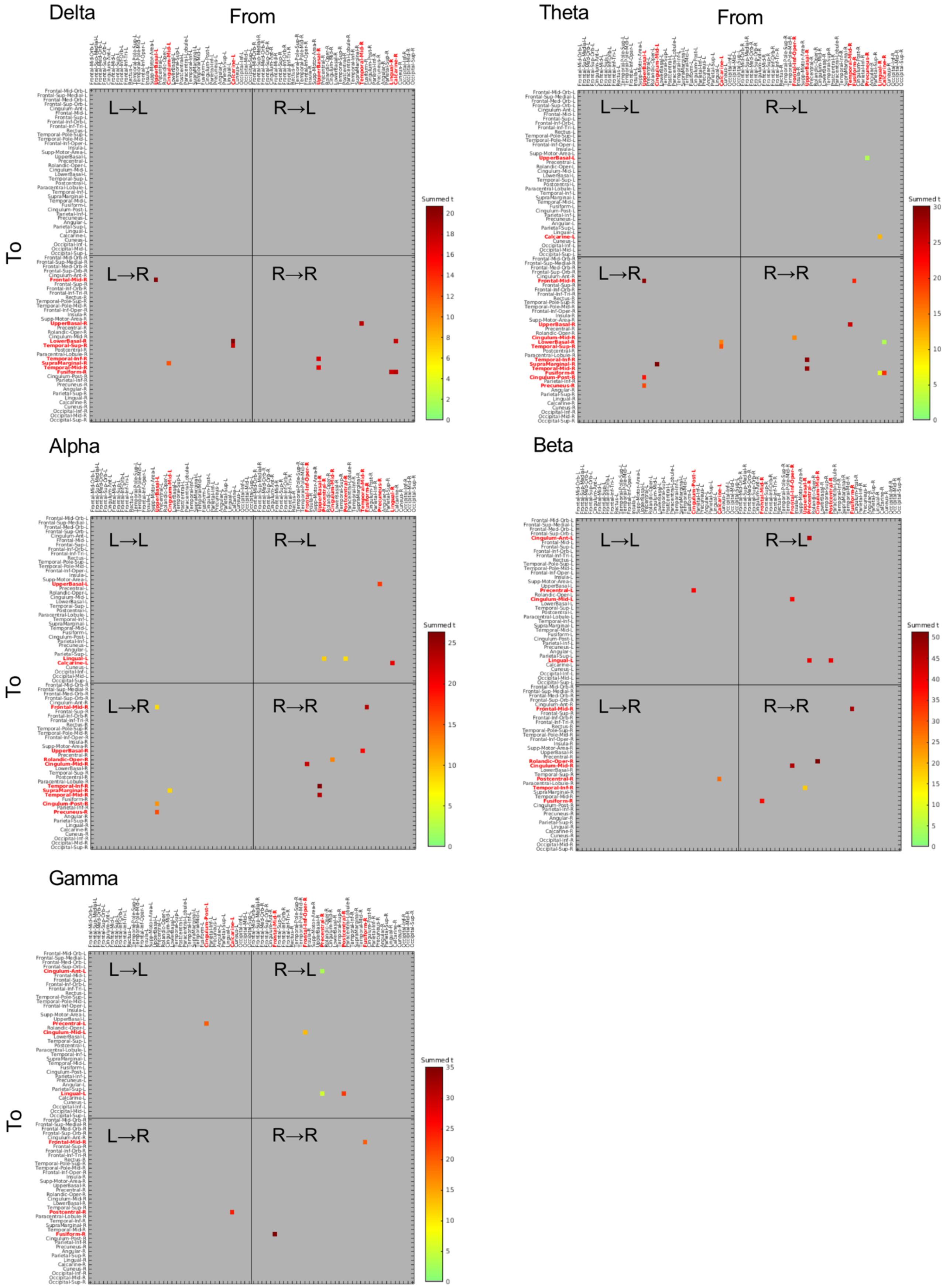

Legend: The group-difference (healthy subjects (N=126) and schizophrenia patients (N=139)) of each EEG band activity ($p < 0.0001$, corrected; two-tailed) is shown for each band EEG activity. The ROI at the top represents the starting point and the ROI at the side represents the ending point.

Abbreviations: L, left; R, right.

**Supplementary Figure 2** Effective connectivity in each EEG band activity in healthy subjects (N=126) and schizophrenia patients (N=139)

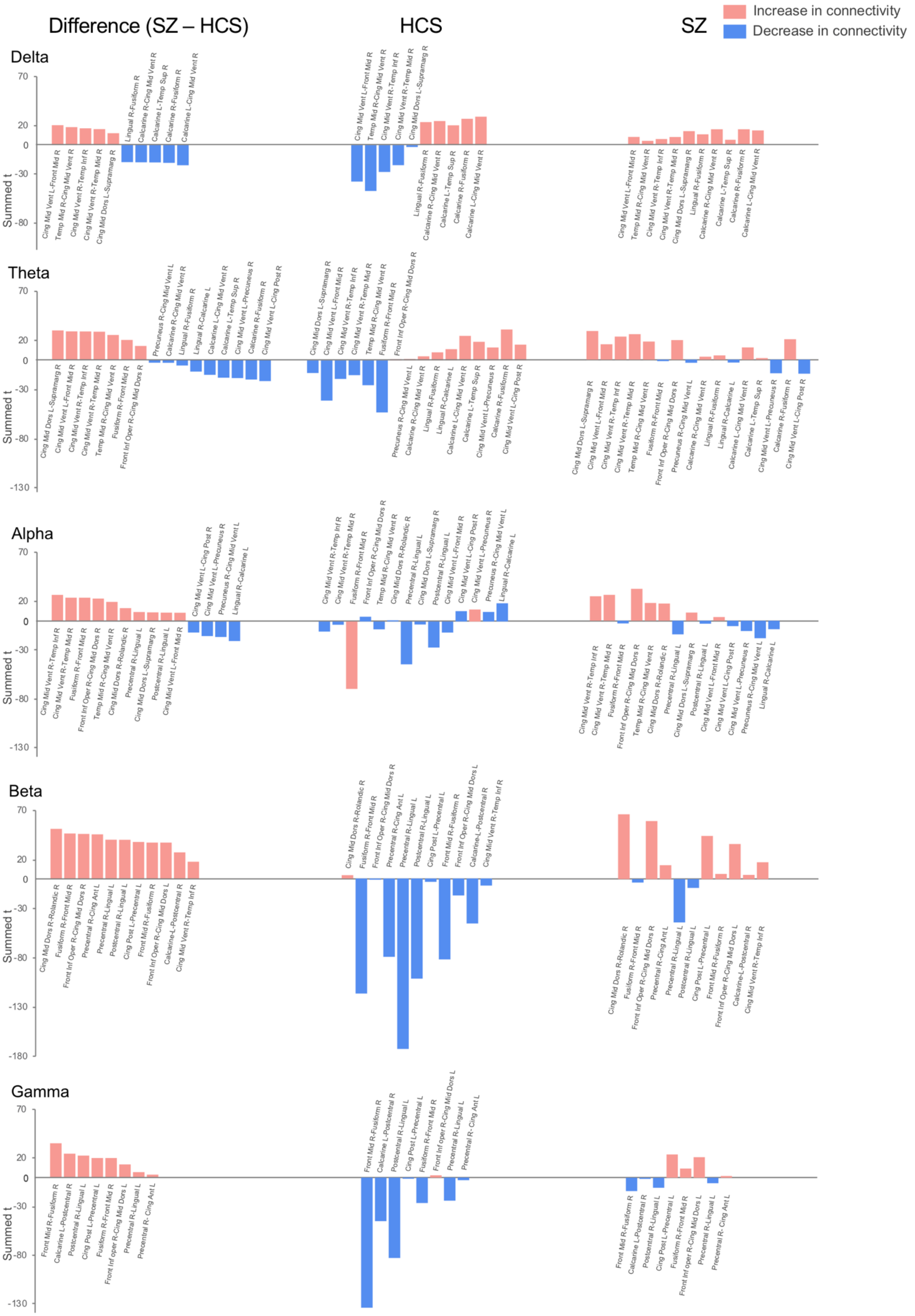

Abbreviations: SZ, schizophrenia; HCS, healthy comparison subject; L, left; R, right; Front Mid, middle frontal; Front Inf Oper, opercular part of inferior frontal; Cing Ant, anterior cingulate; Cing Mid Dors, dorsal middle cingulate; Cing Mid Vent, ventral middle cingulate; Cing Post, posterior cingulate; Temp Sup, superior temporal; Temp Mid, middle temporal; Temp Inf, inferior temporal; Rolandic, Rolandic operculum; Supramarg, Supramarginal.

**Supplementary Figure 3** Connectivity matrix of 76 × 76 anatomical region of interests (ROIs) for each band EEG activity in healthy subjects (N=126) and schizophrenia patients who did not have either anxiolytics nor anticholinergics (N=80)

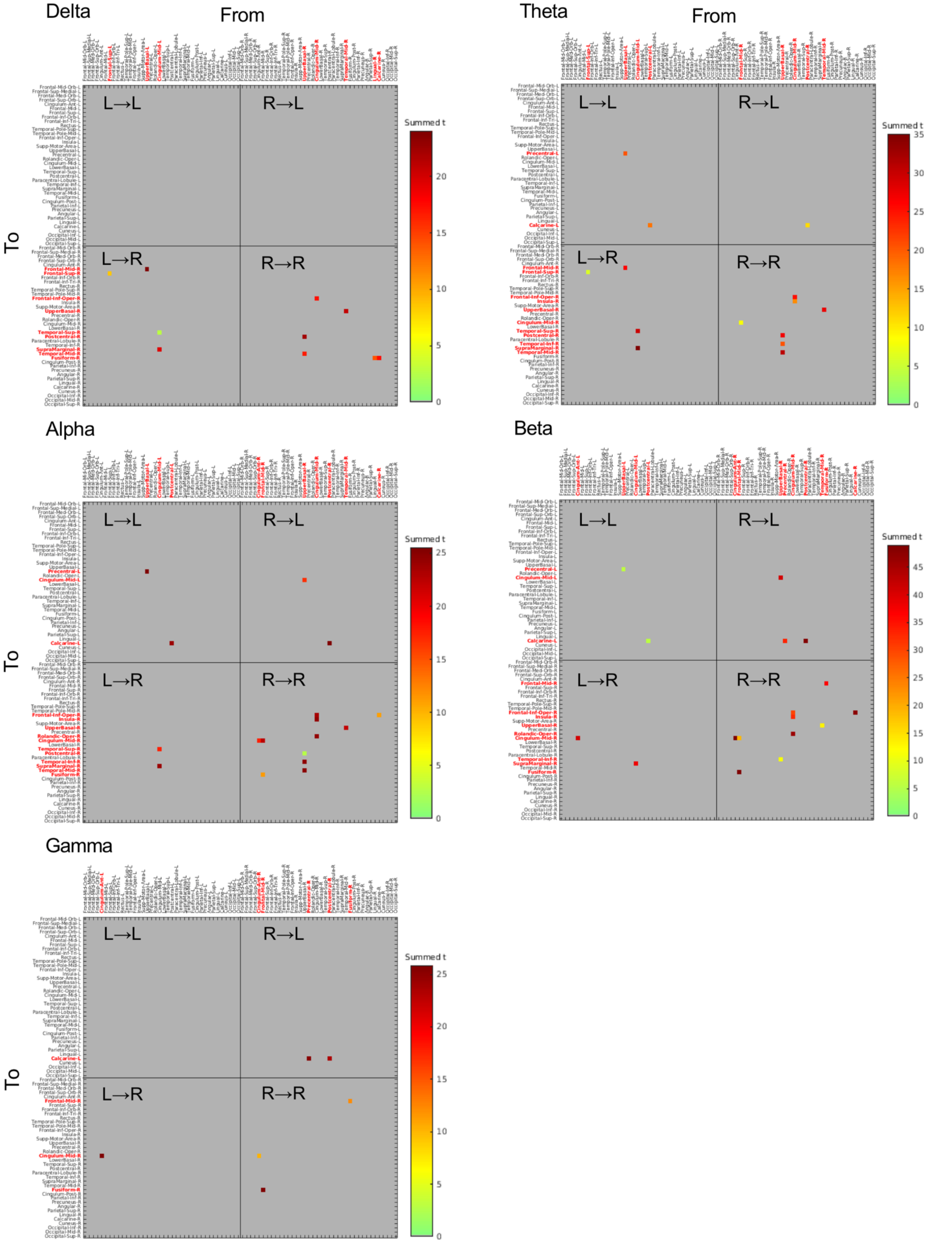

Legend: The group-difference [healthy subjects (N=126) and schizophrenia patients who did not have either anxiolytics nor anticholinergics (N=80)] of each EEG band activity ($p$ < 0.0001, corrected; two-tailed) is shown for each band EEG activity. The ROI at the top represents the starting point and the ROI at the side represents the ending point.

Abbreviations: L, left; R, right.

**Supplementary Figure 4** Effective connectivity in each EEG band activity in healthy subjects (N=126) and schizophrenia patients who did not have either anxiolytics nor anticholinergics (N=80)

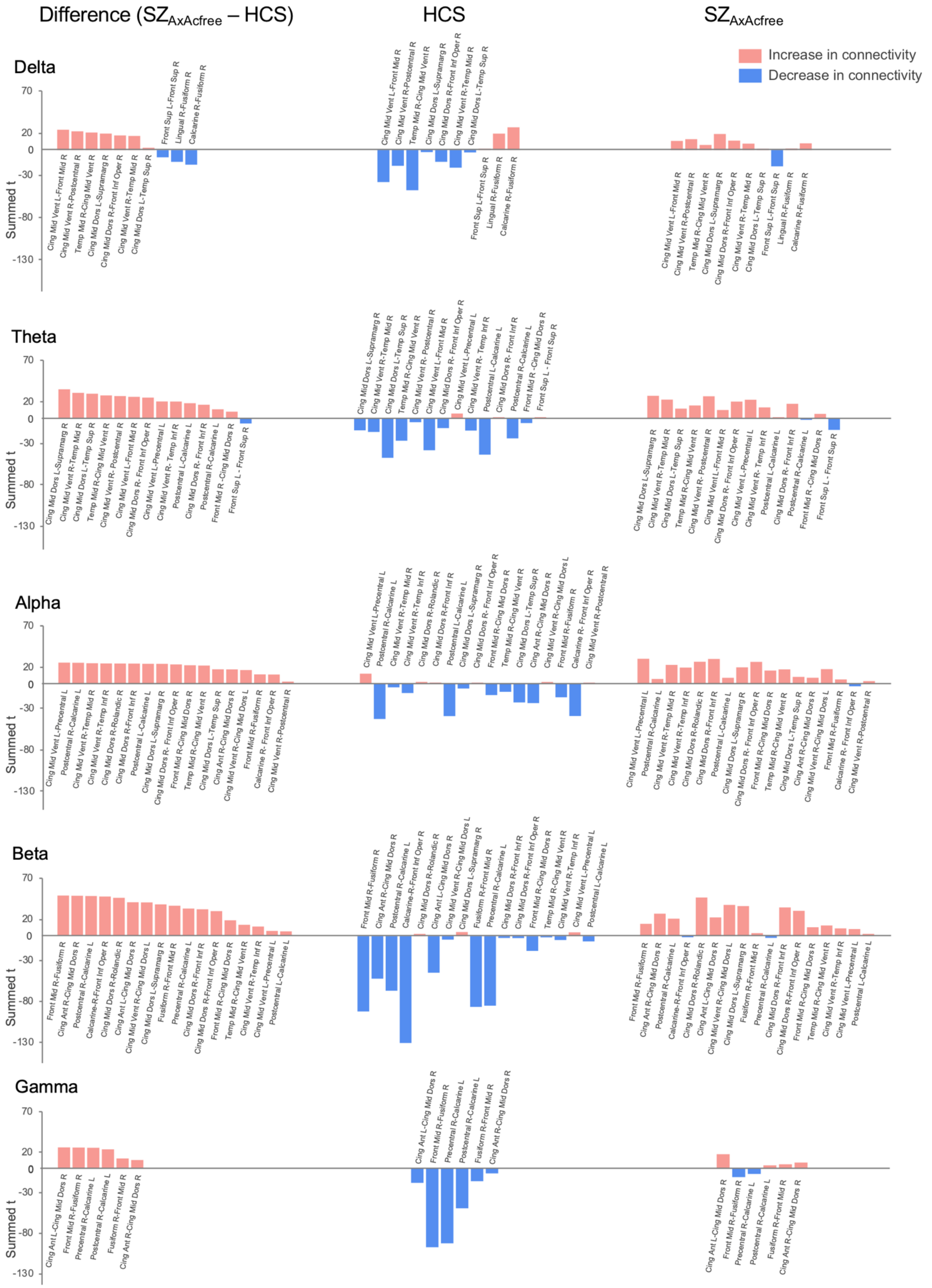

Abbreviations: SZ, schizophrenia; HCS, healthy comparison subject; L, left; R, right; Front Sup, superior frontal; Front Mid, middle frontal; Front Inf, inferior frontal; Front Inf Oper, opercular part of inferior frontal; Cing Ant, anterior cingulate; Cing Mid Dors, dorsal middle cingulate; Cing Mid Vent, ventral middle cingulate; Temp Sup, superior temporal; Temp Mid, middle temporal; Temp Inf, inferior temporal; Rolandic, Rolandic operculum; Supramarg, Supramarginal.

**Supplementary Figure 5** Neural networks underlying resting-state EEG activity in schizophrenia patients who did not have either anxiolytics nor anticholinergics (N=80)

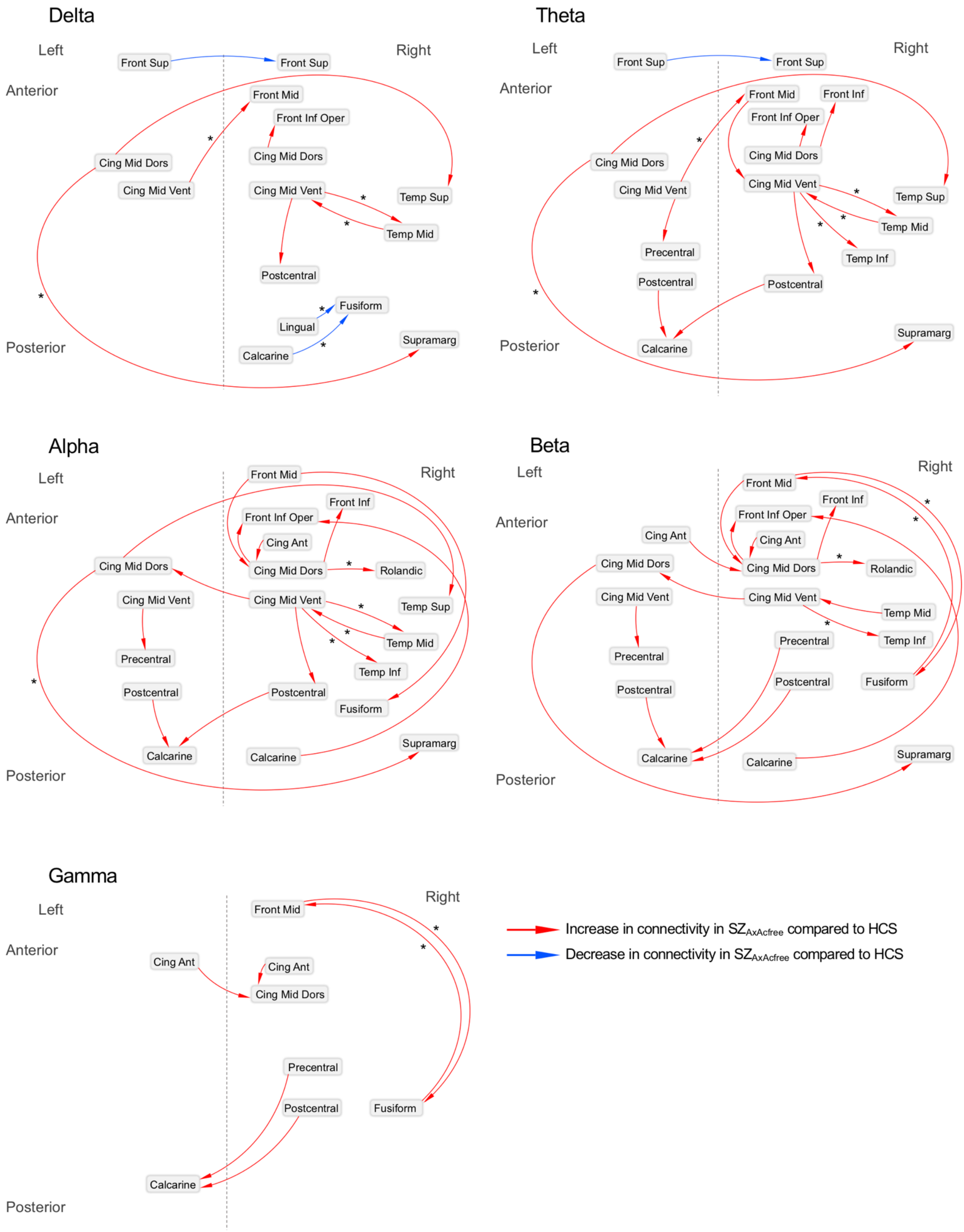

Legends: Difference of effective connectivity between healthy subjects (N=126) and schizophrenia patients who did not have either anxiolytics nor anticholinergics (N=80) is shown; Asterisks indicate the increased or decreased information flows observed in schizophrenia patients (N=139) compared to healthy subjects (N=126).

Abbreviations: SZ, schizophrenia; HCS, healthy comparison subject; L, left; R, right; Front Sup, superior frontal; Front Mid, middle frontal; Front Inf, inferior frontal; Front Inf Oper, opercular part of inferior frontal; Cing Ant, anterior cingulate; Cing Mid Dors, dorsal middle cingulate; Cing Mid Vent, ventral middle cingulate; Temp Sup, superior temporal; Temp Mid, middle temporal; Temp Inf, inferior temporal; Rolandic, Rolandic operculum; Supramarg, Supramarginal.
